# Preparation of R_F_-(VM-SiO_2_)*_n_*-R_F_/AM-Cellu Nanocomposites, and Use Thereof for the Modification of Glass and Filter Paper Surfaces: Creation of a Glass Thermoresponsive Switching Behavior and an Efficient Separation Paper Membrane

**DOI:** 10.3390/polym9030092

**Published:** 2017-03-04

**Authors:** Hideo Sawada, Yuki Suto, Tomoya Saito, Yuri Oikawa, Katsumi Yamashita, Satoshi Yamada, Masashi Sugiya, Jun-ichi Suzuki

**Affiliations:** 1 Department of Frontier Materials Chemistry, Graduate School of Science and Technology, Hirosaki University, Hirosaki 036-8561, Japan; hideosawak@yahoo.co.jp (Y.S.); hideosaw@cc.hirosaki-u.ac.jp (T.S.); h15ds101@hirosaki-u.ac.jp (Y.O.); h16ms321@hirosaki-u.ac.jp (K.Y.); suzuki@kankyokougaku.com (J.-i.-S.); 2 Research and Development Division, Nippon Chemical Industrial Co., Ltd., Koto-ku, Tokyo 136-8515, Japan; satoshi.yamada@nippon-chem.co.jp (S.Y.); masashi.sugiya@nippon-chem.co.jp (M.S.); 3 Kankyo Kogaku Co., Ltd., Hirosaki 036-8093, Japan

**Keywords:** fluoroalkyl end-capped oligomer, theromoresponsive surface, alkyl-modified cellulose, surface modification, LCST behavior, highly oleophobic/superhydrophilic characteristic, superoleophilic/superhydrophobic characteristic, separation of W/O emulsion

## Abstract

Fluoroalkyl end-capped vinyltrimethoxysilane oligomeric silica/alkyl-modified cellulose (AM-Cellu) nanocomposites [R_F_-(CH_2_-CHSiO_2_)*_n_*-R_F_/AM-Cellu; *n* = 2, 3; R_F_ = CF(CF_3_)OC_3_F_7_] were prepared by the sol-gel reactions of the corresponding oligomer [R_F_-(CH_2_-CHSi(OMe)_3_)*_n_*-R_F_] in the presence of AM-Cellu. The nanocomposites thus obtained were applied to the surface modification of glass to exhibit a highly oleophobic/superhydrophilic characteristic on the modified surface at 20 °C. Interestingly, a temperature dependence of contact angle values of dodecane and water was observed on the modified surface at 20~70 °C, and the dodecane contact angle values were found to decrease with increasing the temperatures from 20 to 70 °C to provide from highly oleophobic to superoleophilic characteristics on the surface. On the other hand, the increase of the water contact angle values was observed with the increase in the temperatures under similar conditions to supply superhydrophilic to superhydrophobic characteristics on the modified surface. The corresponding nanocomposites were also applied to the surface modification of the filter paper under similar conditions to afford a superoleophilic/superhydrophobic characteristic on the surface. It was demonstrated that the modified filter paper is effective for the separation membrane for W/O emulsion to isolate the transparent colorless oil.

## 1. Introduction

Cellulose-based materials, such as paper, cloth, and cotton fabrics, play an important role in a variety of fields, such as the textile industry, packaging, printing, and coating areas [1,2,3]. However, cellulose presents a great sensitivity to moisture, quite differently from the traditional plastics, such as polystyrene, poly(vinyl chloride), polypropylene, polyethylene, and poly(methyl methacrylate). Therefore, the transformation of such hydrophilic materials into hydrophobic, especially superhydrophobic derivatives, has been hitherto strongly desirable in order to open a new route to the development of novel cellulose-based materials. In fact, there have been numerous reports on the fabrication of superhydrophobic cellulose materials by using titania/longer alkylated silane coupling agents [4], calcium carbonate/alkyl ketene dimer [5], polydiallyldimethylammonium chloride/silica particles/longer perfluoroalkylated silane coupling agents [6], longer alkylated silane coupling agent/silica nanoparticles [7], polystyrene/PTFE (polytetrafluoroethylene) [8], polymethysiloxane [9,10], graft-copolymerization via atom transfer radical polymerization (ATRP) technique [11,12], and gold nanoparticle immobilization [13]. In the cellulose-based materials, filter paper is widely used for adsorption of liquid, and separation of solids and liquids due to the porous structure constructed by microfibers. Therefore, the exploration of the cellulose derivatives including filter paper possessing a superoleophilic/superhydrophobic characteristic has been of particular interest from the applicable viewpoint of oil/water separation membrane. Hitherto, we have been comprehensively studying the development of the fabrication of superamphiphobic [14,15], superoleophobic/superhydrophilic [16,17], and superoleophilic/superhydrophobic [16,18,19] surfaces by using fluoroalkyl end-capped vinyltrimethoxysilane oligomeric silica composites as a key intermediate. From this point of view, it is very important to apply the corresponding fluorinated oligomeric silica composites to the development of novel cellulose-based materials possessing a unique surface characteristic. Here we report that fluoroalkyl end-capped vinyltrimethoxysilane oligomeric silica/alkyl-modified cellulose (AM-Cellu) nanocomposites can be easily prepared by the sol-gel reaction of the corresponding oligomer in the presence of AM-Cellu. The modified glass surface treated with these fluorinated nanocomposites was found to exhibit a highly oleophobic/superhydrophilic characteristic at 20 °C. However, interestingly, the modified surface treated with the fluorinated nanocomposites was found to provide the switching behavior from highly oleophobic/superhydrophilic to superoleophilic/superhydrophobic characteristics on the surface by increasing the temperatures from 20 to 70 °C. The nanocomposites were also applied to the surface modification of filter paper under similar conditions to supply a superoleophilic/superhydrophobic characteristic on the modified surface. The modified filter paper thus obtained has been applied to the separation membrane for W/O emulsion to isolate the transparent colorless oil. These results will be described in this article.

## 2. Experimental Section

### 2.1. Measurements

Dynamic light scattering (DLS) measurements were measured by using Otsuka Electronics DLS-7000 HL (Tokyo, Japan). Contact angles were measured using a Kyowa Interface Science Drop Master 300 (Saitama, Japan). Field emission scanning electron micrographs (FE-SEM) were obtained by using JEOL JSM-7000F (Tokyo, Japan). Dynamic force microscope (DFM) was recorded by using SII Nano Technology Inc. E-sweep (Chiba, Japan). Optical and fluorescence microscopies were measured by using OLYMPUS Corporation BX51 (Tokyo, Japan).

### 2.2. Materials

Alky-modified cellulose (Metolose-90SH-100^®^) was obtained from Shin-Etsu Chemical Co., Ltd. (Tokyo, Japan) and used as received. Span 80 (sorbitan monooleate) was purchased from Tokyo Chemical Industrial Co., Ltd. (Tokyo, Japan). Fluoroalkyl end-capped vinyltrimethoxysilane oligomer was prepared according to our previously-reported method [20]. Glass plate (borosilicate glass) (micro cover glass: 18 mm × 18 mm) was purchased from Matunami glass Ind. Ltd. (Osaka, Japan) and was used after washing well with 1,2-dichloroethane. Filter paper (Advantec 131) was received from Advantec Toyo Kaisha, Ltd. (Tokyo, Japan).

### 2.3. Preparation of Fluoroalkylated Vinyltrimethoxysilane Oligomeric Silica/AM-Cellu Nanocomposites [R_F_-(VM-SiO_2_)_n_-R_F_/AM-Cellu]

**Scheme 1:**
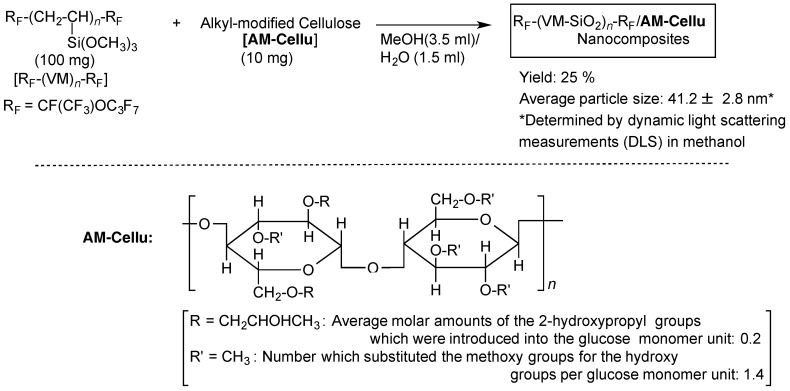
Preparation of R_F_-(VM-SiO_2_)*n*-R_F_/AM-Cellu nanocomposites.

A typical procedure for the preparation of R_F_-(VM-SiO_2_)*_n_*-R_F_/AM-Cellu nanocomposites is as follows: To a methanol solution (3.5 mL) containing fluoroalkyl end-capped vinyltrimethoxysilane oligomers [100 mg; RF-[CH2CHSi(OMe)3]*_n_*-R_F_; R_F_ = CF(CF_3_)OC_3_F_7_; *M*n = 730 (R_F_-(VM)*_n_*-R_F_)] was added aqueous AM-Cellu (10 mg) solution (1.5 mL). The mixture was stirred with a magnetic stirring bar at room temperature for 5 h. Water was added to the obtained crude products after the solvent was evaporated off. The aqueous suspension was stirred with magnetic stirring bar at room temperature for one day. The fluorinated oligomeric silica/AM-Cellu nanocomposites were isolated after centrifugal separation for 30 min. The nanocomposite product was washed well with water several times, and then was dried under vacuum at 50 °C for two days to afford the expected composites as white powders (28 mg) (see Scheme 1).

### 2.4. Surface Modification of Glass Treated with the R_F_-(VM-SiO_2_)_n_-R_F_/AM-Cellu Nanocomposites

The methanol solution (3.5 mL) containing R_F_-(VM)*_n_*-R_F_ oligomer (100 mg) and aqueous AM-Cellu (100 mg) solution (1.5 mL) was stirred with a magnetic stirring bar at room temperature for 5 h at room temperature. The glass plate (18 × 18 mm^2^ pieces) was dipped into this solution at room temperature and left for 1 min. These glass plates were lifted from the solutions at a constant rate of 0.5 mm/min and were left to dry at room temperature for one day; finally, these were dried under vacuum for one day at room temperature to afford the modified glass. The modified filter papers (25 × 25 mm^2^ pieces) were prepared under similar conditions. The contact angles of dodecane and water were measured by the deposit of each droplet (2 μL) on these modified glasses and filter papers, which were left in the box (55 mm × 98 mm × 26 mm) and equipped with a temperature controller after the pre-incubation of these modified ones left in the box at each temperature (20~70 °C) for 1 h.

### 2.5. Preparation of the Surfactant-Stabilized Water in Oil (1,2-Dichloroethane) Emulsion

The surfactant (span 80: 20 mg) was added into the mixture of water (0.05 mL) and 1,2-dichloroethane (5.0 mL). The expected white-colored W/O emulsion was easily prepared through the ultrasonic irradiation of the obtained mixture for 5 min at room temperature.

## 3. Results and Discussion

### 3.1. Preparation of the R_F_-(VM-SiO_2_)_n_-R_F_/AM-Cellu Nanocomposites

It is well-known that the original cellulose has no solubility toward not only water, but also the traditional organic media. However, alkyl-modified cellulose [AM-Cellu] has a good solubility in water and methanol. Thus, we tried to use the AM-Cellu for the nanocomposite reaction with fluoroalkyl end-capped vinyltrimethoxysilane oligomer [R_F_-(VM)*_n_*-R_F_]. In fact, the R_F_-(VM)*_n_*-R_F_ oligomer was found to undergo the sol-gel reaction in the presence of AM-Cellu to afford the corresponding fluorinated oligomeric silica/AM-Cellu composites [R_F_-(VM-SiO_2_)*_n_*-R_F_/AM-Cellu] in 25% isolated yield (see Scheme 1).

The obtained composites in Scheme 1 were found to provide a good dispersibility and stability in methanol. Thus, we have measured the size of these fluorinated composites in methanol by dynamic light-scattering (DLS) measurements at 25 °C. The fluorinated composites are nanometer size-controlled fine particles of 41 nm (number-average diameter), as shown in Scheme 1.

### 3.2. Surface Modification of Glass by Using the R_F_-(VM-SiO_2_)_n_-R_F_/AM-Cellu Nanocomposites

R_F_-(VM-SiO_2_)*_n_*-R_F_ oligomeric nanoparticles, which are prepared by the sol-gel reaction of the corresponding oligomer [R_F_-(VM)*_n_*-R_F_] under alkaline conditions, have already been applied to the surface modification of glass to exhibit an oleophobic/superhydrophobic characteristic on the surface [21]. Thus, we have prepared the modified glasses by using the R_F_-(VM-SiO_2_)*_n_*-R_F_/AM-Cellu nanocomposites illustrated in Scheme 1, and dodecane and water contact angle values on the modified glass surface were measured at 20 °C (the dodecane and water contact angle values on the original glass surface are 0° and 50°, respectively). The results are shown in Table 1.

As shown in Table 1, the fluorinated nanocomposites were found to afford a highly oleophobic characteristic on the modified surface, because the dodecane contact angle value is 69°, and a time dependence was not observed in the dodecane contact angle measurements.

On the other hand, the effective decrease of water contact angle values from 113° to 0° over 25 or 30 min was observed on the modified surface. The smooth flip-flop motion between hydrophobic fluoroalkyl groups and the hydrophilic AM-Cellu moieties in the composites would provide the superhydrophilic surface at the interface with water, and it takes 25 or 30 min to replace the fluoroalkyl groups by the AM-Cellu moieties, adapting itself to an environmental change from air to water on the modified surface. It was previously reported that such smooth flip-flop motion between oleophobic and hydrophilic surfaces can be observed on the modified glass surface treated with the fluoroalkyl end-capped vinyltrimethoxysilane–acryloylmorpholine cooligomer [22] and fluoroalkyl end-capped vinyltrimethoxysilane oligomeric silica/calcium silicide nanocomposites [17].

It is well known that nonionic polysoaps such as poly(*N*-isopropylacrylamide) (PNIPAM) undergo a thermally-induced phase separation in their aqueous solutions when heated above the lower critical solution temperature (LCST) [23]. In our present R_F_-(VM-SiO_2_)*_n_*-R_F_/AM-Cellu nanocomposites, interestingly, the AM-Cellu is also a temperature-sensitive polymers, and can exhibit the LCST around 70 °C in aqueous solutions as shown in Figure S1 in Supplementary Materials, indicating that it gives the transparent aqueous solution below the LCST and occurs the phase separation through the oleophilic-olophilic interaction in the AM-Cellu above the LCST. From this point of view, it is expected that the oleophilic–oleophilic interaction between the oleophilic moieties in the AM-Cellu in the composites and oleophilic compounds such as dodecane should accelerate the flip-flop motion between the fluoroalkyl groups and oleophilic moieties in the composites on the surface at around 70 °C (the LCST of the AM-Cellu) to provide the LCST-like behavior in air through the dodecane contact angle measurements. Thus, we have studied the temperature dependency for the dodecane and water contact angle measurements at 20~70 °C on the modified glass surface treated with the R_F_-(VM-SiO_2_)*_n_*-R_F_/AM-Cellu nanocomposites illustrated in Scheme 1, and the results are also shown in Table 1.

**Figure 1:**
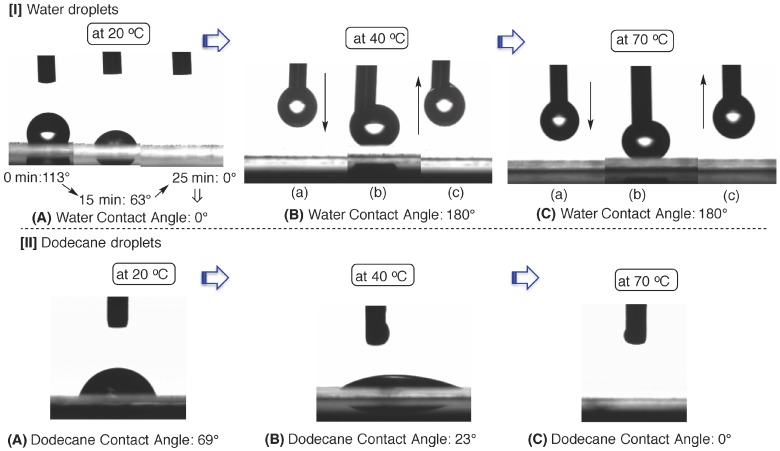
Charge-coupled device camera images of the water and dodecane droplets on the modified glass surfaces treated with the R_F_-(VM-SiO_2_)*n*-R_F_/AM-Cellu nanocomposites.

As shown in Table 1, the dodecane contact angle values were found to decrease with the increase of the temperatures from 20 to 70 °C, providing the switching behavior from higher oleophobic to superoleophilic characteristics on the modified surfaces. On the other hand, the wetting behavior for water on the modified surface has been changed from superhydrophilic to superhydrophobic characteristics by increasing the temperatures from 20 °C to over 40 °C, since the water contact angle values increased from 0° to 180° on the surface under such conditions. In this way, it was verified that the present fluorinated nanocomposites can provide the switching behavior from highly oleophobic/superhydrophilic to superoleophilic/superhydrophobic characteristics on the modified surface by increasing the temperature on the modified surface from 20 to 70 °C. This unique temperature dependence for water and dodecane contact angle values is also illustrated in Figure 1.

We can observe the time dependence for the water contact angle measurements on the modified surface at 20 °C, and the effective decrease of water contact angle value from 113° to 0° over 25 min was observed to exhibit a superhydrophilic characteristic on the surface (see Figure 1**I**(**A**)). On the other hand, interestingly, water contact angle value was found to increase dramatically from 0° to 180° with increasing the temperatures from 20 °C to over 40 °C (see Figure 1**I**(**B**),(**C**)). Additionally, we can observe the water droplet that adhered to the needle tip (see Figure 1**I**(**B**)(**c**)) even after the pull-up process of the needle from the modified surface (Figure 1**I**(**B**)(**b**)), indicating that the water contact angle value is 180°. A similar result was observed in the Figure 1**I**(**C**).

Figure 1**II** shows the smooth decrease of the dodecane contact angle value from 69° to 23°, and finally 0°, with the increase of the temperatures from 20 to 40 °C and, successively, 70 °C. Such superoleophilic characteristic on the modified surface at 70 °C would be due to the effective oleophilic-oleophilic interaction between the oleophilic moieties in the AM-Cellu units in the nanocomposites and dodecane; because the AM-Cellu can give the LCST at around 70 °C as shown in Figure S1 in Supplementary Materials.

**Figure 2:**
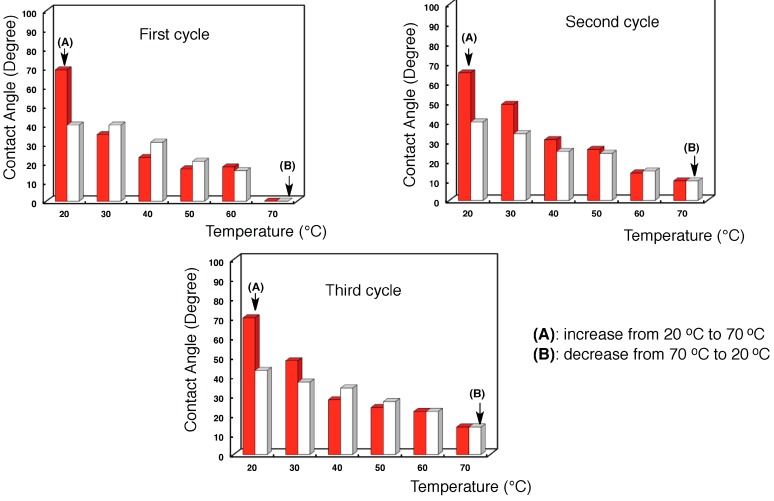
Temperature dependence of the dodecane contact angle values on the modified glass surfaces treated with the R_F_-(VM-SiO_2_)*n*-R_F_/AM-Cellu nanocomposites.

The recyclability of the dodecane contact angle values on the modified glass surface from 20 to 70 °C was evaluated, and the results are shown in Figure 2.

As shown in first cycle in Figure 2(A), dodecane contact angle values were found to decrease from 69° to 0° with increasing the temperatures from 20 to 70 °C. In contrast, interestingly, the dodecane contact angle values were found to increase from 0° to 42° with the decrease of the temperatures from 70° to 20° (see first cycle in Figure 2(B)). The process was additionally repeated two cycles, and the results are shown in second and third cycles in Figure 2, respectively. A similar tendency for the decrease and increase of dodecane contact angle values with the temperature changes was obtained in second and third cycles. However, the similar dodecane contact angle values during 20~70 °C were not observed in each cycle, suggesting that these modified films consist of the cross-linked silica composite networks.

**Figure 3:**
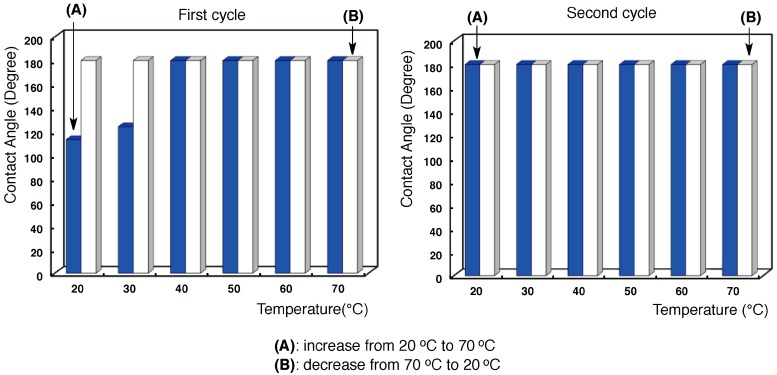
Temperature dependence of the water contact angle values on the modified glass surfaces treated with the R_F_-(VM-SiO_2_)*n*-R_F_/AM-Cellu nanocomposites.

Similarly, the recyclability of the water contact angle values on the modified glass surface was evaluated, and the results are shown in Figure 3.

**Figure 4:**
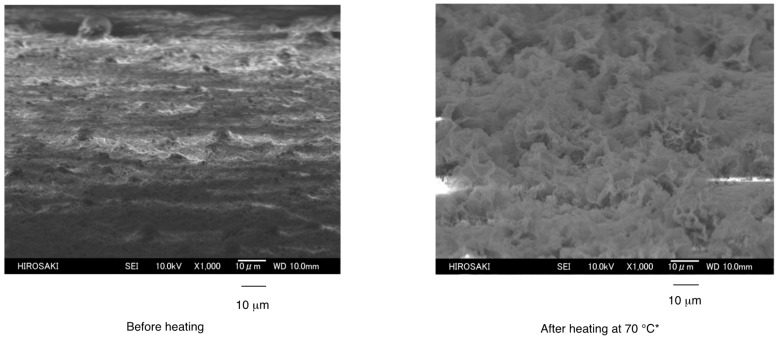
FE-SEM images of the modified glass surface treated with the R_F_-(VM-SiO_2_)*n*-R_F_/AM-Cellu nanocomposites at 20 °C (before heating) and after heating at 70 °C*. * The modified glass was used for the measurements after the decrease of the temperature from 70 to 20 °C.

**Figure 5:**
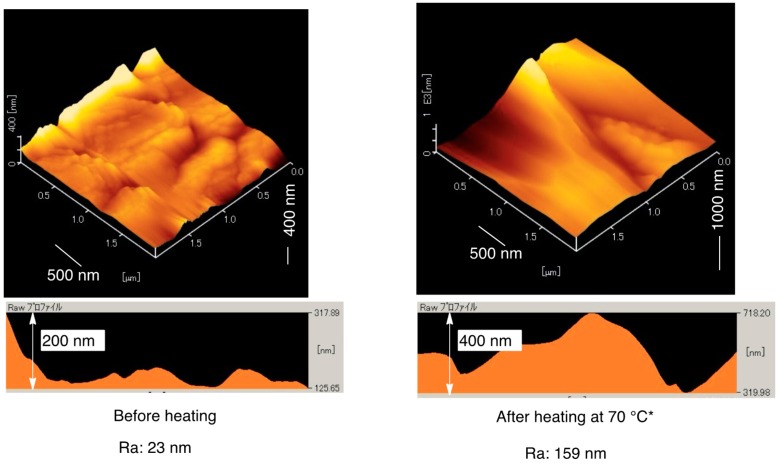
DFM (dynamic force microscopy) topographic images of the modified glass surface treated with the R_F_-(VM-SiO_2_)*n*-R_F_/AM-Cellu nanocomposites at 20 °C (before heating) and after heating at 70 °C*. * The modified glass was used for the measurements after the decrease of the temperature from 70 to 20 °C.

Water contact angle values were found to increase from 113° to 180° with increasing the temperatures from 20 °C to over 40 °C (see first cycle in Figure 3(A)). In fact, as shown in Figure 4, the higher roughness architecture was observed on the modified glass surface after heating at 70 °C, compared with that before heating. In addition, Figure 5 shows that the topographical image of the modified glass surface after heating at 70 °C can provide a higher roughness property (the roughness average: Ra = 159 nm) than that (Ra = 23 nm) before heating. These findings also suggest that such higher Ra value would be derived into the superhydrophobic surface toward the modified glass surface after heating.

The decrease of the water contact angle values was not observed at all with the decrease of the temperatures from 70 to 20 °C as shown in first cycle in Figure 3(B). The temperature dependency of the water contact angle values was not observed at all in second cycle (or third cycle: data not shown) under similar conditions, keeping the constant value: 180° for the increase or the decrease process of the temperatures (see the second cycles in Figure 3(A),(B)). This finding would be due to the fluorinated oligomeric silica network structures on the modified surfaces.

### 3.3. Surface Modification of Filter Paper by Using the R_F_-(VM-SiO_2_)_n_-R_F_/AM-Cellu Nanocomposites

**Figure 6:**
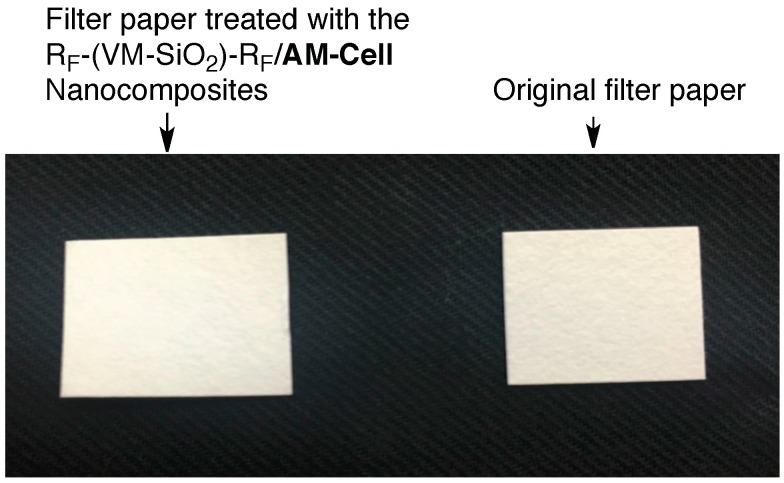
Photograph of the modified filter paper treated with the R_F_-(VM-SiO_2_)-R_F_/AM-Cell nanocomposites and the original filter paper.

Next, we tried to have the surface modification of filter paper by using the R_F_-(VM-SiO_2_)*_n_*-R_F_/AM-Cellu nanocomposites illustrated in Scheme 1. As shown in Figure 6, we have succeeded in preparing the uniformly modified filter paper by using the R_F_-(VM-SiO_2_)*_n_*-R_F_/AM-Cellu nanocomposites, quite similar to that of the original filter paper. Moreover, it was clarified that the adhesion ability of the modified filter paper surface is strong enough, and we can keep the appearance of the modified surface even after rubbing the modified surface with the finger, due to the presence of the AM-Cellu units in the nanocomposites. In contrast, the adhesion ability of the modified filter paper treated with the original R_F_-(VM-SiO_2_)*_n_*-R_F_ oligomeric nanoparticles is poor, and the corresponding particles were easily released from the modified filter paper after rubbing the surface with a finger.

The modified filter paper treated with the R_F_-(VM-SiO_2_)*_n_*-R_F_/AM-Cellu nanocomposites was found to exhibit a superoleophilic/superhydrophobic characteristic on the modified filter paper surface, because the dodecane and water contact angle values at 25 or 70 °C are 0° and 180° (Table 2), respectively; although both of the dodecane and water contact angle values on the original filter paper are 0° under similar conditions.

Unexpectedly, we cannot observe the temperature dependency for these contact angles at all with the increase or decrease of the temperatures during 20 to 70 °C, and the same contact angle values were observed in each case.

**Figure 7:**
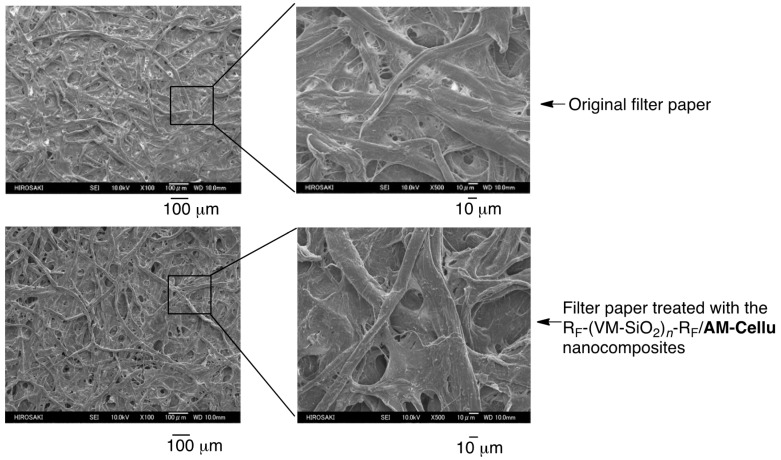
FE-SEM images of the modified filter paper surface treated with the R_F_-(VM-SiO_2_)*n*-R_F_/AM-Cellu nanocomposites.

**Figure 8:**
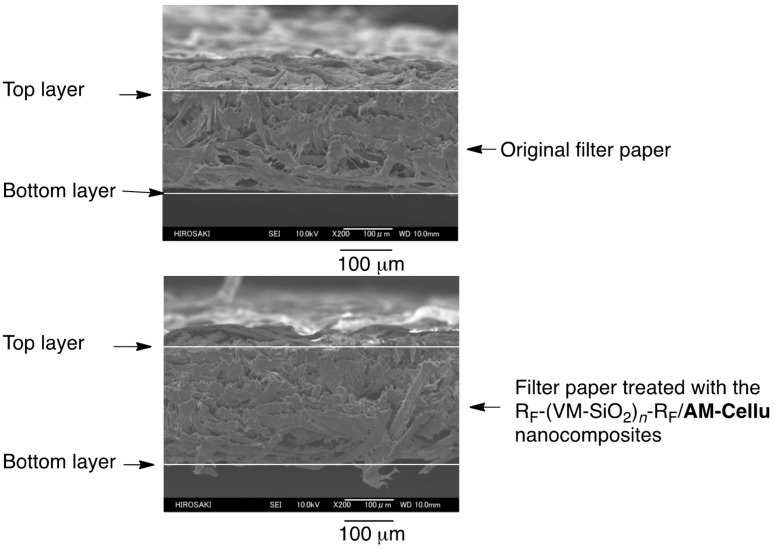
FE-SEM images of the cross-section of the modified filter paper surface treated with the R_F_-(VM-SiO_2_)*n*-R_F_/AM-Cellu nanocomposites.

Creation of superhydrophobic surface is, in general, realized by enhancing the surface roughness [24,25,26]. Thus, we have studied on the surface roughness of the modified filter papers treated with the R_F_-(VM-SiO_2_)*_n_*-R_F_/AM-Cellu nanocomposites illustrated in Scheme 1 by using FE-SEM measurements. The original filter paper was also studied under similar conditions, for comparison. These results are shown in Figure 7 and Figure 8.

As shown in Figure 7, we can observe the similar roughness on the modified filter paper surface to that of the original filter paper, and the surface appearance is quite similar to that of the original one. In addition, the similar FEM-SEM image of the cross-section of the modified filter paper to that of the original one was observed (see Figure 8).

**Figure 9:**
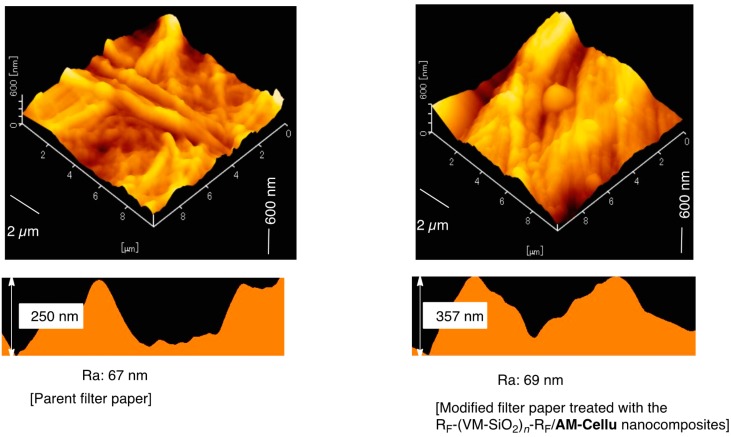
DFM topographic images of the parent filter paper surface and the modified filter paper surface treated with the R_F_-(VM-SiO_2_)*n*-R_F_/AM-Cellu nanocomposites.

We have also studied the DFM (dynamic force microscopy) measurements of the modified filter paper, including the original filter paper, and the results are shown in Figure 9.

As shown in Figure 9, unexpectedly, the modified filter paper surface was found to exhibit the similar topographical image of that of the original filter paper, and the roughness average values (Ra: 69 nm) of the modified filter paper was also the same as that (Ra: 67 nm) of the original one.

We can observe the formation of the clear void moieties on the modified filter paper surface (see Figure 7), and such void moieties should interact smoothly with oil droplets to afford the superoleophilic characteristic on the modified surface due to the lower surface tension of oils than that of water. On the other hand, the architecture of the similar roughness surface for the modified filter paper to that of the original filter paper suggests that the surface modification of the filter paper would be due to the two-dimensional modification, providing the thin coating fluorinated nanocomposite surface; because the R_F_-(VM)*_n_*-R_F_ oligomer should undergo the smooth sol-gel reaction in the presence of the AM-Cellu under non-catalytic conditions to supply the two-dimensional cross-linked silica network structures on the surface. Thus, such smooth roughness surface would provide the superhydrophobic characteristic on the modified surface related to the presence of the longer fluoroalkyl groups in the nanocomposites.

Heretofore, there have been numerous reports on the creation of the superhydrophobic/superoleophilic filter paper through the architecture of the roughness surface by using a variety of methods, such as a porous film formation composed of poly(tetrafluoroethylene) nanoparticles [8], spray coating with hydrophobic silica nanoparticles suspension [7], the treatment with a mixture of hydrophobic silica nanoparticles and polystyrene solution in toluene [27], and two-step dip coating, which consists of the first dip coating of calcium carbonate and, subsequently, the coating with alkyl ketene dimer [5]. These superhydrophobic surfaces are in general realized by enhancing the surface roughness. However, FE-SEM and DFM measurements illustrated in Figure 7 and Figure 9 show that the surface morphology of our present superoleophilic/superhydrophobic surface is quite similar to that of the parent filter paper, and the enhancement of the surface roughness was not observed during the surface modification process. We believe that this surface modification method, without the enhancement of the surface roughness for affording the superhydrophobic characteristic, is the first example.

### 3.4. Separation of W/O Emulsion by Using the Modified Filter Paper Treated with the R_F_-(VM-SiO_2_)_n_-R_F_/AM-Cellu Nanocomposites as the Separation Membrane

**Figure 10:**
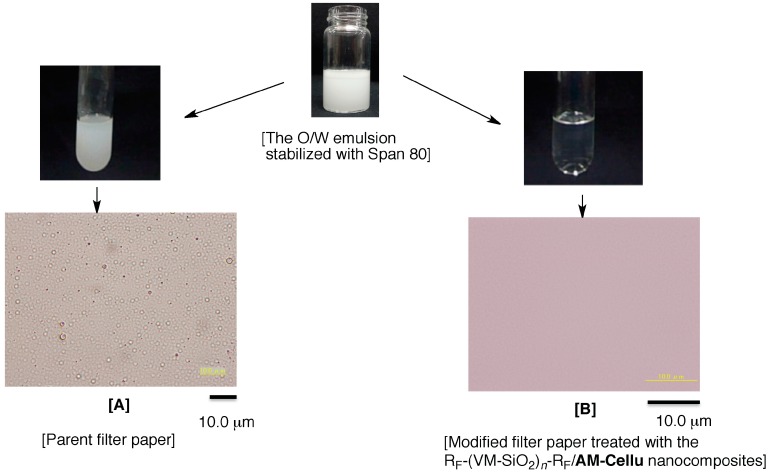
Photograph of optical microscopy of the isolated oil after the separation of the W/O emulsion by using the parent filter paper and the modified filter paper treated with the R_F_-(VM-SiO_2_)*n*-R_F_/AM-Cellu nanocomposites.

The superoleophilic surface has, in general, a strong affinity toward oils. Thus, the surfaces possessing the superoleophilic/superhydrophobic characteristic can simultaneously repel water and strongly absorbs oils. Such behavior should be applicable to the oil/water separating materials [5,7,28,29]. Thus, we tried to separate the water-in-oil (W/O) emulsion by using the modified filter paper treated with the R_F_-(VM-SiO_2_)*_n_*-R_F_/AM-Cellu nanocomposites as the separation membrane. The surfactant (span 80: 20.0 mg)-stabilized water (0.05 mL)-in-oil (1,2-dichloroethane: 5.00 mL) (W/O) emulsion was prepared under ultrasonic conditions for 5 min at room temperature. The original non-treated filter paper was also used as the separation membrane under similar conditions, for comparison. These results are shown in Figure 10.

The original filter paper was not effective for the membrane to separate the W/O emulsion under reduced pressure conditions. However, interestingly, it was demonstrated that the present modified filter paper is effective for the separation of the W/O emulsion, and only transparent colorless oil has been isolated under reduced pressure. In fact, as shown in Figure 10B, the optical micrograph shows that the water droplet cannot be detected at all in the isolated colorless oil, although we can easily detect the water droplet in the case of the use of the original filter paper (see Figure 10A).

We tried to study the reusability of the modified filter paper as the separation membrane, and the colorless oil was quantitatively isolated under similar conditions even after the use of the W/O emulsion three times as the following recovered ratios: first time, 95%; second time, 94%; and third time, 95%.

In this way, the present R_F_-(VM-SiO_2_)*_n_*-R_F_/AM-Cellu nanocomposites may be developed in the new separation membrane for the mixture of oil and water in a wide variety of fields.

## 4. Conclusions

In summary, we have developed a simple strategy for the creation of a novel thermoresponsive surface by using fluoroalkyl end-capped vinyltrimthoxysilane oligomeric silica/AM-Cellu nanocomposites [R_F_-(VM-SiO_2_)*_n_*-R_F_/AM-Cellu]. The resulting surfaces are sensitive to temperature changes. Especially, it was demonstrated that the modified surface treated with the R_F_-(VM-SiO_2_)*_n_*-R_F_/AM-Cellu nanocomposites can afford from highly oleophobic/superhydrophilic to superoleophilic/superhydrophobic characteristics on the modified glass surface corresponding to the temperature changes from 20 to 70 °C. Interestingly, such fluorinated nanocomposites were applied to the surface modification of filter paper to provide a superoleophilic/superhydrophobic characteristic on the modified surface. More interestingly, the modified filter paper possessing a superoleophilic/superhydrophobic characteristic was also applied to the membrane for the separation of the W/O emulsion to isolate the colorless oil. Therefore, our present fluorinated nanocomposites have high potential for the novel separation membrane of the mixtures of oil and water in a wide variety of fields, including the area of organic synthesis.

## Figures and Tables

**Table 1:** Temperature dependence for the contact angles of dodecane and water on the modified glass surface treated with the R_F_-(VM-SiO_2_)-R_F_/AM-Cellu nanocomposites.

Temperature (°C)	Contact angel (°)							
	Dodecane ^a^	Water						
		Time (min)						
		0	5	10	15	20	25	30
**20**	**69**	**113**	**95**	**88**	**63**	**38**	**0**	**0**
**30**	**35**	**124**	**96**	**86**	**75**	**51**	**17**	**0**
**40**	**23**	**180**	**- ^b^**	**- ^b^**	**- ^b^**	**- ^b^**	**- ^b^**	**- ^b^**
**50**	**17**	**180**	**- ^c^**	**- ^c^**	**- ^c^**	**- ^c^**	**- ^c^**	**- ^c^**
**60**	**18**	**180**	**- ^c^**	**- ^c^**	**- ^c^**	**- ^c^**	**- ^c^**	**- ^c^**
**70**	**0**	**180**	**- ^c^**	**- ^c^**	**- ^c^**	**- ^c^**	**- ^c^**	**- ^c^**

^a^ A time dependence for the dodecane contact angle measurements was not observed; ^b^ water contact angle was 180° in each time; ^c^ water contact angle measurement was not completed due to the vaporization of water droplet.

**Table 2:** Dodecane and water contact angle values on the modified filter paper treated with the RF-(VM-SiO2)n-RF/AM-Cellu nanocomposites at 25 and 70 °C.

Temperature	25 °C	70 °C
Dodecane contact angle:	0°	0°
Water contact angle:	180°	180°

## Supplementary Materials

The supplementary materials are available online at www.mdpi.com/2073-4360/9/3/92/s1. Figure S1: Temperature dependence of transmittance at 500 nm of aqueous solutions of the AM-Cellu (40 g/dm^3^).
