# Supplementary material for: Preparation of RF-(VM-SiO2)n-RF/AM-Cellu Nanocomposites, and Use Thereof for the Modification of Glass and Filter Paper Surfaces: Creation of a Glass Thermoresponsive Switching Behavior and an Efficient Separation Paper Membrane
Source: Polymers (Basel). 2017 Mar 4;9(3):92. doi: 10.3390/polym9030092 (PMC6432376; doi:10.3390/polym9030092)
Supplement: Supplementary file 1 [file polymers-09-00092-s001.docx]

Supplementary Materials: Preparation of R_F_-(VM-SiO_2_)*_n_*-R_F_/AM-Cellu Nanocomposites, and Use Thereof for the Modification of Glass and Filter Paper Surfaces: Creation of a Glass Thermoresponsive Switching Behavior and an Efficient Separation Paper Membrane

Hideo Sawada, Yuki Suto, Tomoya Saito, Yuri Oikawa, Katsumi Yamashita,
Satoshi Yamada, Masashi Sugiya and Jun-ichi Suzuki

**Figure S1.** Temperature dependence of transmittance at 500 nm of aqueous solutions of the AM-Cellu (40 g/dm^3^).
